# Sex differences in opioid analgesia and addiction: interactions among opioid receptors and estrogen receptors

**DOI:** 10.1186/1744-8069-9-45

**Published:** 2013-09-08

**Authors:** Cynthia Wei-Sheng Lee, Ing-Kang Ho

**Affiliations:** 1 Center for Drug Abuse and Addiction, China Medical University Hospital, 2 Yuh-Der Road, Taichung 40447, Taiwan; 2 China Medical University, Taichung, Taiwan; 3 Graduate Institute of Clinical Medical Science, China Medical University, Taichung, Taiwan; 4 Center for Neuropsychiatric Research, National Health Research Institutes, Zhunan, Miaoli County, Taiwan

**Keywords:** Sex differences, Opioid analgesia, Opioid addiction, Opioid receptors, Estrogen receptors

## Abstract

Opioids are widely used as the pain reliever and also notorious for being addictive drugs. Sex differences in the opioid analgesia and addiction have been reported and investigated in human subjects and animal models. Yet, the molecular mechanism underlying the differences between males and females is still unclear. Here, we reviewed the literature describing the sex differences in analgesic responses and addiction liabilities to clinically relevant opioids. The reported interactions among opioids, estrogens, opioid receptors, and estrogen receptors are also evaluated. We postulate that the sex differences partly originated from the crosstalk among the estrogen and opioid receptors when stimulated by the exogenous opioids, possibly through common secondary messengers and the downstream gene transcriptional regulators.

## Review

### Introduction

Opioids are potent analgesics used to treat acute and chronic pain, and also notorious for their potential to cause addiction [1-4]. Gender differences in the experience of clinical and experimental pain [5-7] and the susceptibility to opioid addiction [8] have been reported. General observations suggest that there are more adult men than women involved in illicit drug abuse [9]. However, this contrasts to the clinical and animal studies indicating that females are more susceptible to drug abuse problem than males [10]. Besides the sociocultural factors, there must be true differences between the biological differences that influence drug abuse and pain perception, and estrogen has been proposed to be one of the key players [11,12].

### Sex differences in opioid analgesia and addiction

Population-based studies suggest that women are more likely to experience chronic pain syndromes and report more severe pain at a higher frequency than men [13-19]. Human studies indicate that females and males have similar thresholds for cold and ischemic pain [20,21], while pressure pain thresholds are lower in females than males [22,23]. Females tolerate less thermal pain (cold, heat) and pressure than males [24-26], but this is not the case for tolerance to ischemic pain, which is comparable in both genders [27,28]. Based on a review of the available literature published between 1966 and 1998, Miaskowski and Levine suggest that opioids are better analgesics for women [29]. A Chinese population study conducted in southern Taiwan also shows that females consume significantly less morphine via patient-controlled analgesia than males during the first three postoperative days [30]. However, the majority of more recent studies comparing gender report that the potency and efficacy of morphine administered systemically is higher in males than in females against a variety of nociceptive modalities [31-33]. The controversy might be due to that earlier studies did not correct for the body weight differences between men and women. In addition, there are sex differences in reporting pain and seeking pain relief, and health care providers make unwarranted psychogenic attributions regarding pain in female but not male [7,34-36].

A profile of a heroin-addiction epidemic showed that 74 percent of the addicts are males [37]. In the United States, the past year and life time rates of heroin use are higher among men (men = 0.2% vs. women = 0.1%; 2.3% vs. 0.8%, respectively), while equivalent rates of men and women are reported to inject heroin (42.0% vs. 40.7%) [8]. Among adolescent drug users administrated during 2002–2003 in the National Survey on Drug Use and Health, females are 3.91 times more likely to inject heroin than males [38]. Gender differences in the clinical profiles of opioid-dependent individuals have been observed in substance use severity, craving, medical conditions, and impairment in associated areas of functioning. Craving for opioids is significantly higher among women, and women have higher drug, employment, family, medical, and psychiatric Addiction Severity Index composite scores [8]. Among patients entering the maintenance program in Italy, there seems to be an emerging pattern of males who tend to use heroin as their opiate of choice, and are more likely to combine it with cannabis, while females are more likely to using street methadone, with adjunctive use of ketamine, benzodiazepines, hypnotic drugs and/or amphetamines [39]. Moreover, women are at higher risk of abusing opioids through initial prescription painkiller use, and later resort to street methadone to cope with prescription pain killer addiction [39]. Analysis from the U.S. indicates that opioid-addicted women work less and use more cocaine than their male counterparts [40]. The use of drugs of abuse in women may be influenced by psychosocial and hormonal factors, such as psychiatric comorbidity (a higher rate of anxiety disorders) [41-44], more distressing drug-related environment, lower rate of antisocial personality traits [45], and estrogen-regulated neuroendocrine functions [12,39,46]. Sex differences in opioid analgesia and addiction in human and animals have been investigated extensively, and clinically-relevant representative studies are listed in Tables 1 and 2. Effects of opioids are inconsistent among different studies and species, which might result from different genetic backgrounds, ages of the subjects, doses of the opioids used, and assays or end points of the measurements.

**Table 1:** Sex differences in opioid analgesia and addiction in human

**Opioid**	**Receptor**	**Model**	**Effect**	**Reference**
Buprenorphine	ORL1 agonist	Postoperative pain	M < F	[47-49]
	MOR partial agonist			
	KOR antagonist			
Butorphanol	MOR partial agonist	Acute injury	M = F	[50]
	KOR agonist	Thermal, pressure, and ischemic pain (experimental)	M = F	[51]
		Postoperative dental surgery	M < F	[52]
		Cold-water stimulus (experimental)	M > F	[53]
Fentanyl	MOR agonist	Postoperative pain	M < F	[54]
			M = F	[55]
Ketobemidone	MOR agonist	Postoperative pain	M = F	[56]
	NMDA antagonist			
Methadone	MOR agonist	Cancer pain	M = F	[57]
Morphine	MOR agonist	Acute injury	M > F	[50]
	KOR agonist	Thermal, pressure, and ischemic pain (experimental)	M = F	[51]
	DOR agonist			
		Postoperative pain	M > F	[32,33]
			M = F	[58-60]
			M < F	[30,61-64]
Nalbuphine	KOR agonist	Postoperative pain	M = F	[65]
	MOR antagonist			
		Postoperative dental surgery	M < F	[52,66]
Pentazocine	KOR agonist	Acute pain (experimental)	M = F	[67,68]
	MOR partial agonist		M < F	[69]
		Postoperative dental surgery	M < F	[70]
Pethidine	MOR agonist	Postoperative pain	M = F	[60,71]
	KOR agonist			
Heroin	MOR agonist	Addiction epidemic	M > F	[8,37]
	KOR agonist	Adolescent drug users	M < F	[38]
	DOR agonist			

**Table 2:** Sex differences in opioid analgesia and addiction in animals

**Opioid**	**Receptor**	**Species**	**Model**	**Effect**	**Reference**
Buprenorphine	ORL1 agonist	Rat	Hot plate	M = F	[72]
	MOR partial agonist		Tail withdrawal	M > F	[73-75]
	KOR antagonist				
				M = F	[72]
			Temporal summation (thermal stimulus / tail withdrawal)	M > F	[76]
Butorphanol	MOR partial agonist	Rat	Capsaicin-induced hyperalgesia (Tail withdrawal)	M = F	[77]
	KOR agonist				
			Temporal summation (thermal stimulus / tail withdrawal)	M > F	[76]
Fentanyl	MOR agonist	Rat	Tail flick	M = F	[78]
Methadone	MOR agonist	Rat	Tail flick	M > F	[79]
Morphine	MOR agonist	Rat	Abdominal constriction	M > F	[80,81]
	KOR agonist		Hot plate	M > F	[81]–[86]
	DOR agonist			M < F	[87]
			Tail flick	M > F	[76,81,88-94]
				M = F	[95]
			Tail withdrawal	M > F	[74,75,85,86]
			Temporal summation (thermal stimulus / tail withdrawal)	M > F	[96]
		Mouse	Hot plate	M > F	[97-99]
			Tail Flick	M > F	[100]
			Tail Withdrawal	M > F	[101,102]
				M = F	[101]
				M < F	[101]
Nalbuphine	KOR agonist	Rat	Tail withdrawal	M > F	[74,103,104]
	MOR antagonist				
Heroin	MOR agonist	Rat	Acquisition of self-administration	M < F	[105-107]
	KOR agonist				
	DOR agonist				

Factors contributing to sex differences in drug abuse include pharmacokinetics, behavioral phenotypes for drug abuse vulnerability, sensitivity to aversive properties of drugs, puberty and adolescence, and genetic factors beyond hormones as reviewed by Wetherington [108]. Given the ubiquitous actions and gender differences of sex hormones in the central nervous system, many investigators have attempted to relate sex differences in opioid analgesia to gonadal hormone levels [73,80-82,88-93,100,109-118]. Yet, the neurological and cellular mechanisms underlying the sexually dimorphic analgesic and addictive responsiveness to opioids remain poorly understood [31].

### Estrogen regulation of opioid receptors

The analgesic effects and addiction liability of opioids are mediated by opioid receptors. Based on the molecular and pharmacological properties, three conventional opioid receptors – μ (MOR), δ (DOR), and κ (KOR) – have been characterized [119]. A non-opioid branch of opioid receptors, opioid receptor-like 1 (ORL1) receptor, also known as the nociceptin/orphanin FQ peptide (NOP) receptor, has also been identified and displays pharmacological profiles distinct from those of conventional opioid receptors [120]. Activation of opioid receptors inhibits (acute) / superactivates (chronic) adenylate cyclase (AC) activity [121], impedes N- and L-type Ca^2+^ channels, increases phospholipase C activity, activates inwardly rectifying K^+^ channels, and turns on mitogen-activated protein kinases (MAPK) [122,123].

Estrogens, besides the well-established effects on female reproductive functions, exert various actions on the nervous system influencing pain sensation, mood, susceptibility to seizures, and neuroprotection against stroke damage and Alzheimer’s disease [124]. Ovarian steroids have been found to modulate the activity of opioid receptors in healthy women and migraine sufferers [125], and replacement therapies through estrogens and progestagens could restore the activity of central opioid tonus in migraine patients [125]. Estrogen has also been demonstrated to decrease the secretion of β-endorphin, an endogenous opioid peptide, from the Ishikawa cells, an endometrial carcinoma cell line, in a concentration- and time-dependent manner [126]. The spinal KOR and DOR, but not MOR, activity is required for opioid-mediated elevations in maternal nociceptive thresholds, indicating the ability of estrogen to modulate spinal opioid antinociceptive activity [127].

**Figure 1:**
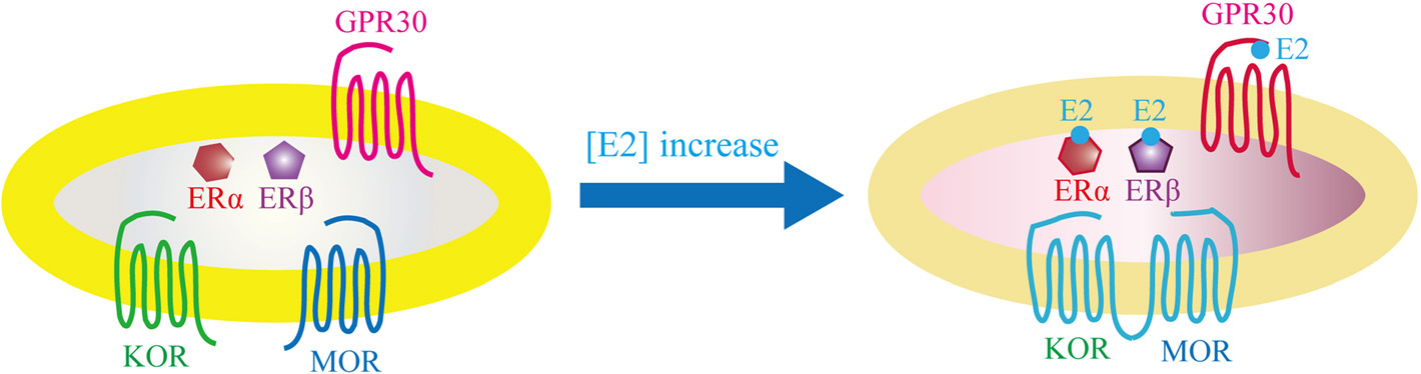
**Schematic representation of the facilitation of KOR/MOR heterodimerization by E2.** Biochemical and behavioral experiments suggest that ERs work cooperatively to increase KOR/MOR expression. We postulate that E2 triggers a signaling complex containing one or multiple ERs, which via an unknown mechanism enhances the formation of KOR/MOR heterodimers and thereby creates the sex difference in opioid actions. Modified from [131].

Sexually dimorphic KOR-mediated antinociception has been demonstrated in antithetical antinociceptive/nociceptive responsiveness of female vs. males to KOR agonists-antagonists [128]. Compared to men, women reported greater analgesic effects from the mixed MOR/KOR ligands: pentazocine, nalbuphine and butorphanol [52,66]. In contrast, selective KOR agonists produced greater antinociceptive effects in male than female animals [129]. An animal study demonstrated that spinal morphine antinociception in females requires concomitant activation of MOR and KOR, and the expression of MOR/KOR heterodimers is more prominent in the spinal cord in females than males [130]. The same group further demonstrated that blockade of coexpressed ERα and GPR30, two types of estrogen receptors (detailed in the following section), substantially decreased MOR/KOR and eliminates mediation by KOR of spinal morphine antinociception, suggesting MOR/KOR could serve as a molecular target for analgesia in women [131] (Figure 1).

17β-estradiol (E2), the major ligand of estrogen receptors during reproductive years, rapidly attenuates the ability of μ-opioids to hyperpolarize guinea pig hypothalamic (β-endorphin, an opioid peptide) neurons. E2 does not compete for MOR or alter the affinity of MOR, but binds to a specific receptor that activates PKA to rapidly uncouple MOR from its K^+^ channel [132]. Increased PKA activity maintains cellular tolerance to MOR agonists in the hypothalamic arcuate nucleus (ARC) neurosecretory cells caused by chronic morphine treatment. Moreover, acute E2 and chronic opioid treatment attenuate MOR-mediated responses via a common PKA pathway [133]. Based on the high density of MOR, but the lack of effects of estrogen on [^35^S]GTPγS binding, it is concluded that MOR interaction with its G-protein is not the target of estrogen’s actions [134]. E2 may modulate the behavioral effects of cocaine by regulating MOR and KOR signaling in mesocorticolimbic brain structures in female rats [135]. In addition, sex-dependent differences have been found in the intake of ethanol in the absence of β-endorphins in mice [136], and in the regulation of gonadal hormone, DOR binding, and MOR density in the hippocampus by prenatal exposure to morphine in rats [137,138].

Multiple antinociceptive assays demonstrated that male rats are markedly more sensitive to morphine analgesia than females [128]. The difference cannot be attributed to gender-linked differences in serum levels of morphine after its injection [81], the acute effects of steroids [81], the pharmacokinetics of morphine [83], MOR number and the binding affinity of the MOR agonists [139], and morphine stimulation of G protein determined using GTPase and [^35^S]GTPγS binding assays [139]. It is postulated that the organizational effects of steroids during critical periods in development, which determine gender-related distinctions, may be significant in the male–female differences [81]. Another explanation for this gender difference is that pathways downstream of MOR and G protein are more efficient in male rats than in female rats such that there is a larger receptor reserve for morphine-mediated antinociception [139]. One mystery that remains poorly understood is that many aspects of sexually dimorphic opioid responsiveness in humans are opposite to that observed in laboratory animals [128].

### Opioid regulation of estrogen receptors

Estrogens act on two types of receptors, nuclear estrogen receptors (ERα and ERβ) and the membrane-associated estrogen G protein-coupled receptor (GPR30, also known as GPER). ERα and ERβ modulate the long-lasting effect of estrogen by regulating gene transcription, whereas GPR30 produces more rapid effects by generation of the secondary messengers and activation of receptor tyrosine kinases [140].

Estrogen promotes the growth and development of breast cancer via ER. ERα is the major ER in neoplastic breast epithelium, whereas ERβ is the predominant ER in normal breast tissue [141,142]. The MOR agonist morphine promotes tumor neovascularization in E2-dependent human breast tumor xenograft model, MCF-7 cell, in mice leading to increased tumor progression at medically relevant concentrations [143]. In contrast, the opioid receptor antagonist naloxone inhibits MCF-7 breast cancer growth in mice [144,145]. Naloxone modulates ERα activity directly as well as indirectly via MOR, suggesting that naloxone-like compounds can be developed as novel therapeutic molecules for breast cancer therapy [145]. Additionally, ERβ is expressed in human vascular endothelial cells, and morphine down-regulates this receptor as determined by real-time RT-PCR [146]. The DOR agonist SNC80 decrease anxiety- and depression-like behavior following withdrawal from chronic cocaine use in male rats [147], and may serve as a potential anxiolytic in females [148]. Further research focusing on the contribution of circulating hormones and DOR agonists on cocaine withdrawal-induced anxiety in females and understanding the sex differences is needed.

The regulatory actions of opioids on estrogen receptors have been described in breast cancer, yet never been linked to the sex differences in opioid analgesia and addiction. Significance of such opioid actions in the sex difference remains elusive, and may be explored both *in vitro* and *in vivo*. The *in vitro* assays can be done by applying the opioids to neuronal cells expressing specific estrogen receptors to characterize the cellular responses of the estrogen receptors. The *in vivo* assays measuring the extent of opioid analgesia and addiction in estrogen receptor knockout mice, with females of different stages of estrous cycle and males, should be performed. Specific antagonists to the opioid receptors should be applied to characterize the interacting opioid receptors.

### Interactions among opioid and estrogen receptors

MOR internalization is correlated with MOR-mediated inhibition of lordosis [149]. MOR antagonists block receptor internalization and facilitate lordosis [149,150]. ERα, but not ERβ, is required for estrogen-induced MOR internalization, suggesting that ERα can mediate rapid actions of estrogen [151]. The mRNA of the ORL1 receptor, the non-canonical member of the opioid receptor family, is present in majority of ERα and/or ERβ mRNA-containing neurons, and the sex-related differences in the ORL1 gene expression in the trigeminal nucleus caudalis appear to be determined in part by estrogen levels [152].

**Figure 2:**
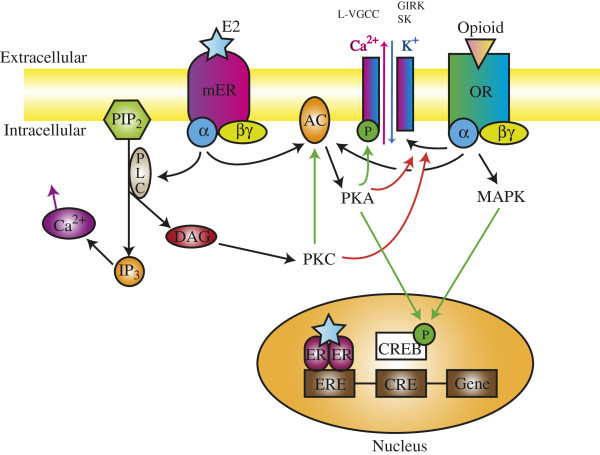
**Diagram of the postulated cross**-**talk between estrogen and opioid receptors.** Upon binding of the opioids, opioid receptors (OR) activate different intracellular signaling pathways through the G protein (composed of α, β and γ subunits). The activation of phospholipase C (PLC) catalyzes the hydrolysis of membrane-bound phosphatidylinositol 4,5-bisphosphate (PIP_2_) into inositol 1,4,5-trisphosphate (IP_3_) and diacylglycerol (DAG). IP_3_ induces calcium release from the endoplasmic reticulum that activates calcium-dependent signaling. DAG activates protein kinase C (PKC). PKC activates adenylate cyclase (AC), which increases cAMP production, and subsequently stimulates protein kinase A (PKA). PKA can phosphorylate various proteins including ion channels (L-type voltage-gated Ca^2+^ channels [L-VGCC], G protein-coupled inwardly rectifying K^+^ channels [GIRK], and small conductance Ca^2+^-dependent K^+^ channels [SK]) and cAMP-responsive element binding protein (CREB). The activation of the mitogen-activated protein kinase (MAPK) transduction cascades can stimulate multiple targets, including nuclear transcription factors (such as CREB), cytoplasmic enzymes (including tyrosine hydroxylase), cytoskeletal proteins, and ion channels. Estradiol (E2) can activate the membrane-bound estrogen receptor (mER) and modulate the ionic conductance through phosphorylation of ionotropic receptors or uncoupling of OR from their ionic channels or intracellular effectors. E2 can also bind to nuclear ER dimers and thereby bind to the estrogen-responsive element (ERE) on the DNA, resulting in the activation of specific gene transcription. Additionally, rapid effects of E2 mediated by mER can lead to CREB phosphorylation, altering gene transcription through the interaction with the cAMP responsive element (CRE). Modified from [181].

GPR30, the plasma membrane ER, is expressed in pain-relevant areas of the rat central nervous system, and the expression levels are similar in the male and female [153-156]. GPR30 activation leads to hyperalgesia in rats [157,158] and spinal nociception in mice [159], and is involved in mediating the rapid pronociceptive effects of E2 [155,157,160]. The downstream mechanisms involve cytosolic calcium increase [161,162], ROS accumulation [163], and neuronal membrane depolarization [159]. Stimulation of plasma membrane ERs is coupled to the activation of the same signaling molecules that participate in most membrane initiated signaling cascades as opioid receptors, e.g., protein kinase A, protein kinase B, protein kinase C, phospholipase C, inositol triphosphate, MAPK, ERK, tyrosine kinases, etc. [164-180] Due to the overlapping of the secondary messenger pathways, activation of GPR30 by estrogen is postulated to influence the signaling cascades of the opioid receptors, leading to the sex differences in the effects of opioids because of different GPR30 expression patterns between males and females (Figure 2).

Although opioids and estrogen can activate common signaling pathways, there is no direct evidence that signaling crosstalk among estrogen and opioid receptors contributes to the sex differences in opioid analgesia and addiction. This data gap should be filled by performing assays measuring the extent of opioid analgesia and addiction in opioid receptor knockout mice, with males and females of different stages of estrous cycle. Specific antagonists to the estrogen receptors are required to identify the interacting estrogen receptors in the behavioral assays.

## Conclusions

Although numerous reports have addressed gender differences of opioid receptor agonists, very few directly examined the mechanism. It has been proposed that differences in opioid receptor levels, distribution and efficiency of signaling and neural circuitry modulated by opioid receptor activation cause the sexual dimorphism [129]. However, direct evidence of the interactions among estrogen and opioid receptors is lacking. Animals deficient of estrogen receptors ERα, ERβ, or GPR30 lack the estrogen-regulated opioid effects, and hence display distinct analgesic and addictive responses to morphine. Functional interactions between estrogens and opioids should be investigated to provide the insight into gender differences in analgesia and addiction at both cellular and physiological levels. Male sex hormone such as testosterone may also play a role in opioid analgesia and addiction, as anabolic androgenic steroids have been shown to alter opioid receptor expression in SH-SY5Y human neuroblastoma cells [182]. This review focuses on estrogen receptors, but does not exclude the possibility that androgen receptors could cross-talk with opioid receptors and thereby contribute to the sex differences of opioid effects. Organismal factors must be considered when interpreting the data, since just as a male is not a female, a mouse is not a small rat, and a primate is not a human. Developmental stages, drug doses, routes of drug administration, types of assays employed, and genetic backgrounds should be considered and matched in future randomized clinical studies to define the sex differences in opioid analgesia and addiction.

## Abbreviations

AC: Adenylate cyclase; DOR: δ-opioid receptor; E2: 17β-estradiol; ERα: Estrogen receptor α; ERβ: Estrogen receptor β; GPR30/GPER: Estrogen G protein-coupled receptor; KOR: κ-opioid receptor; MAPK: Mitogen-activated protein kinases; MOR: μ-opioid receptor; NOP: Nociceptin/orphanin FQ peptide; ORL1: Opioid receptor-like 1 receptor.

## Competing interests

Only the authors listed are responsible for the content and preparation of this manuscript. The authors declare no conflict of interest.

## Authors’ contributions

CW-SL drafted the manuscript and reviewed the literature. I-KH designed the review topic and helped write the manuscript. Both authors read the approved the final manuscript.
